# Preparation of Rat Brain Aggregate Cultures for Neuron and Glia Development Studies

**DOI:** 10.3791/1304

**Published:** 2009-09-30

**Authors:** Hisami Koito, Jianrong Li

**Affiliations:** Department of Veterinary Integrative Biosciences, Texas A & M University

## Abstract

An in vitro system that recapitulates the development and differentiation of progenitors into mature neurons and glia in the central nervous system (CNS) would provide a powerful platform for neuroscientists to investigate axo-glial interactions, properties and differentiation of multipotent progenitors, and progression of oligodendroglial lineage cells at the cellular and molecular level.  We describe here a CNS aggregate culture system from embryonic rat forebrains, which can be maintained in a serum-free medium up to 3-4 weeks and is used in our laboratory as a model to study neuron-glia interaction and CNS myelination.  This video clip will demonstrate how to isolate and grow these CNS aggregate cultures from E16 rat brain.  Furthermore, from the same brain dissection, highly enriched regular dissociated neuronal cultures can be readily obtained and used for various studies on CNS neurons or used for co-cultures with other cells.

**Figure:**
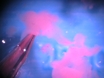


## Protocol

### Preparation before dissection

*Surgical tools:* sterilize all dissection scissors and forceps by autoclave

*Coverslip cleaning:*

Soak all coverslips (15mm in diameter for 24-well plate) in 1L grass beaker in 33% HCl for at least 24 h
Wash with running water for 10 min with occasional shake to remove all residual HCl
Rinse with Millipore water
Drain water off
Soak coverslips in 95-98% ethanol
Transfer coverslips to a clean tissue on a flat plate and air dry the coverslips
Transfer coverslips to a glass beaker, cover with aluminum foil and autoclave *Note: *Coverslips can be stored at this stage
Place individual coverslip into culture plates. Ready for coating

*Coverslip coating:*

Dilute 100 x stock of poly-D-lysine (PDL, 10 mg/ml in 0.5% bovine serum albumin in PBS, stored as aliquots at -20°C) with PBS and filter-sterilize (0.22 μm)
Coat coverslips with 1 x PDL solution (~ 0.15 ml/cm^2^) for 2 h at 37°C in an incubator
Remove PDL solution and wash 3 times with sterile ddH_2_O and dry coverslips completely

*Note: *Coat enough coverslips/plates for each dissection. PDL-coated coverslips can be stored for a few weeks at 4°C.

### Media and solutions

*Dissection medium (DM): *Use sterile ice cold Hank's Balanced Salt Solution (w/o Ca^2+^, Mg^2+^) (HBSS, Invitrogen 14175) supplemented with 10 mM HEPES (Invitrogen 15630).

*Aggregate culture media:* The DMEM/NBB27 medium contains DMEM/Neurobasal (1:1 vol:vol), 2% B27, 1 x Sato, 0.5 mM sodium pyruvate, 0.75mM GlutaMAX, 60μg/ml N-acetylcysteine, 5 μg/ml insulin, 10nM d-Biotin and 1% penicillin/streptomycin. To make 100 ml of the aggregate culture medium, mix 50 ml DMEM (w/o pyruvate/glutamine, Invitrogen 11960), 50 ml Neurobasal medium (Invitrogen 211034), 375 μl GlutaMax (100 x, Invitrogen 35050), 5.5 mg sodium pyruvate (Sigma P2256), 2 ml B27 (Invitrogen 17504), 6.3 mg N-acetylcysteine (Sigma A8199), 1 ml of Sato stock (100 x stock, see below), 25 μl of d-Biotin stock (4000 x stock, 40 μM in PBS stored as aliquots at -20°C.  Sigma B4639), 100 μl of insulin (1000 x stock, 5 mg/ml in 0.01N HCl stored as aliquots at -20°C, Sigma 16634), and 1 ml penicillin/streptomycin (100 x stock, Invitrogen 15140), filter-sterilize and store at 4°C.

*Sato 100 x stock solution:* To make 40 ml of the stock: mix 40 ml Neurobasal with 400 mg apo-transferrin (Sigma T2252), 400 mg BSA (Sigma A9647), 10 μl of progesterone (25 mg/ml ethanol, stored as aliquots at -20°C. Sigma P8783), 64 mg putrescine (Sigma P7505), and 40 μl of sodium selenite (30 μM in PBS, stored as aliquots at -20°C, Sigma S5261). Filter sterilize the Sato stock solution, and store as aliquots at -20°C. 

*Neuron plating medium (PM* *):* Neurobasal Medium, 2% B27, 2mM Glutamine (100x stock, aliquots stored at -20°C, Invitrogen 25030), 25μM glutamic acid (100  stock, aliquots stored at -20°C) and 1% penicillin/streptomycin. 

*Neuron culture medium (CM* *):* Neurobasal Medium, 2% B27, 2mM Glutamine (100x stock, aliquots stored at -20°C) and 1% penicillin/streptomycin. 

*5-FdU stock:* 100x stock, 1mM 5-fluoro-2'-deoxyuridine (Sigma F0503) and1mM uridine (Sigma U3003) in Neurobasal medium. Filter sterilized and stored as aliquots at -20°C.

*Papain digestion solution (make fresh prior to dissection):*

Dissolve 3.2 mg L-Cysteine (Sigma C-7352) in 4ml DM
Adjust pH to around 7.4 with 1N NaOH (test on pH test strips) and place in water bath at 37°C *Note: *Perform the next step right before tissue digestion (see below).
Add papain to a final concentration of 20 unit/ml
Filter sterilize (0.22mm), and place in water bath at 37°C

*Trypsin inhibitor solution (make fresh prior to dissection):*

Dissolve 0.2 g trypsin inhibitor (Sigma T7295) in 20ml DM
Check pH and adjust pH to ~7.4 with 1 N NaOH
Filter sterilize, and place in water bath at 37°C

### Brain Dissection

*Setup:*

Sterilized forceps and scissors
70% ethanol
Clean tissue paper and diaper pad on bench
10cm Petri dishes with ice-cold DM for uterus and fetus on bench
Ice platform at dissection microscope
2-3 6cm dishes with DM on ice
Warm PM in a 37°C water bath

Euthanize a pregnant Sprague-Dawley rat (E16) according to a procedure approved by your Institutional Animal Care and Us Committee (IACUC)
Lay the rat on the diaper pad and spay alcohol on the abdominal region
Use tweezers to hold the abdominal skin and a pair of scissors to make a V shape incision cutting only the skin
Spread apart the skin and use another sterilized tweezers and scissors to cut through the muscle layer
Extract the chain of the embryo sacs and transfer them into a 10cm dish containing ice-cold DM
Using a microdissection scissors, carefully remove each embryo from the sac and the brain from each embryo, and place freed brains into a clean 10cm dish with DM on an ice platform
In a Laminar hood and under a dissection microscope, remove midbrain/hindbrain sections, and use fine forceps to remove meninges, and transfer the cleaned cortices/hippocampus to a new 6cm dish with DM on an ice pack *Note: *at this stage, the tissue is ready for enzyme digestion.
Make papain and trypsin inhibitor solution and filter sterilize
Remove excess DM from cortices/hippocampi
Add 4ml of the prepared papain digestion solution
Transfer all content to a 50ml tube and place the Falcon tube in a water bath at 37°C for exactly 5 min
Remove papain solution with pipette
Add 5ml trypsin inhibitor solution, swirl the tube, and place in a water bath at 37°C for 2-3 min
Remove trypsin inhibitor solution
Repeat step 13 and 14 three times
Add 20ml warm PM
Triturate until all clumps have disappeared (~20-30 times)
Centrifuge at 100xg for 7min
Resuspend the pellet in 10ml PM and wash twice by centrifugation
Resuspend the pellet in 10ml PM for regular neuron cultures or in NBB27/DMEM for aggregate cultures
Pass the cells through a 70μm cell sieves
Count live cells
Proceed to aggregate cultures as described below or plate the cells for regular neuron cultures *Note: *At this step, highly enriched neuron cultures can be achieved by plating the dissociated cells onto PDL-coating plates at density of 200-640 cell/mm^2^ depending on the purpose of experiments. After cells have attached (~ 2-4h), replace medium with warm PM. If culturing for extended periods of time, on day 4, replace half of the medium with warm CM. For highly purified neuronal cultures, mitotic inhibitor 5-FdU (final concentration 10 μM) can be added at DIV2 to inhibit non-neuronal cell proliferation (i.e., during DIV2-3). After recovery in CM for 2 days, cells are pulse treated again with 5-FdU for another 2 days (DIV 6-7) followed by a complete medium change. Thereafter, replace half of the medium with fresh, warm CM every 3-4 days. Neurons are viable for up to 4 weeks.

### Aggregates preparation and plating

Suspend dissociated cells in NBB27/DMEM medium containing 1x Sato, 10ng/ml CNTF and 10μM forskolin at a density of 2x10^6^ cells/ml
Transfer 2ml of cell suspension into each well of a uncoated 6-well plate
Culture for 3 nights. Each day, gently resuspend the cells once using a P1000 Pipetman*Note: *Cells start to form aggregates(Figure 1).
On the day before aggregate plating, coat plate/coverslips with Matrigel:
  Dilute stock Matrigel (growth factor reduced, BD Biosciences 354230) 1:20 with cold DMEM on ice *Note: *Matrigel aliquots should be prepared per manufacturer protocol. All tips, Eppendorf tubes and solutions for making aliquots of the stock should be cold to avoid Matrigel polymerization. Aliquots of Matrigel are kept at -80°C and thawed at 4°C.
  Coat previously PDL-coated coverslips with 300μl/well of the diluted Matrigel solution overnight in a 37°C incubator
  Wash with warm PBS, then wash plate with warm sterile ddH_2_O and leave the plates in the incubator.
3 days after aggregate formation, gently resuspend cells again and sieve the suspension through a 200μm mesh into a 50ml Falcon tube. Allow aggregates to settle to the bottom of the tube by gravity (~3-5min)
Carefully remove supernatant
Add medium, gently resuspend the aggregates and let them settle again. Repeat this procedure several times to remove dead cells, individual cells and debris. Use microscope to check whether the supernatant still contain non-aggregated cells and debris
Gently resuspend the aggregates in the same NBB27/DMEM medium (Figure 1) and count aggregates
Adjust the density of aggregates to around 25-30 aggreggats/50ul
Transfer aliquots (500-1000μl) of the aggregate suspension to 2ml Eppendorf tubes for plating *Note: *Since aggregates tend to settle down to the bottom of tubes, it is crucial to make multiple tubes of the aggregate suspension for plating. Gently resuspend the aggregates often before seeding them onto coated coverslips or culture plates to ensure similar number of aggregates plated on each coverslip.
Take out the culture plate containing Matrigel-coated coverslips from incubator. Remove solution from each well, wash the coverslips with warm PBS and ddH_2_O, and then dry briefly.  They are now ready for aggregate seeding
Invert a tube containing aggregate suspension to allow them to be distributed evenly before seeding. Load 50μl of aggregate suspension into the center of each coverslip, and gently put the plate back to incubator without any further disturbance to allow aggregates to attach evenly to the coverslip *Note: *The density of aggregates is crucial. If aggregates are seeded too close to each other or if the seeding density is high, they tend to merge together as they grow.
Add additional DMEM/NBB27medium (500 μl) to each well 4-6 h later or early the next morning
To maintain the aggregate culture, half change the medium every 3-4 days. When change medium, be careful not to disrupt the aggregates and this is done by gently adding medium along the side wall of the well. *Note: *Few hours after aggregates attached to the plate, neurite outgrowth from the aggregates can be observed. Axons grow rapidly in the first two weeks and form abundant connections between aggregates (Figure 2). Glial progenitors migrate radially out of the aggregates and differentiate over time into astrocytes and mature oligodendrocytes.

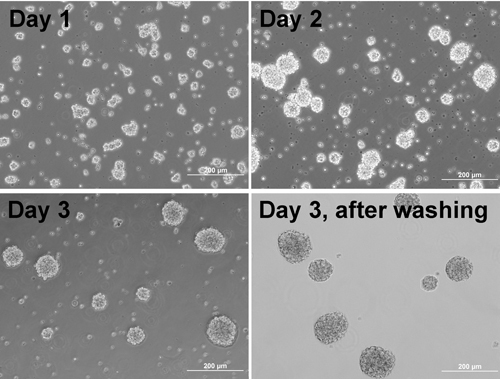
 **Figure 1.** Phase contrast images of  cells forming aggregates at different days and aggregates after washing.


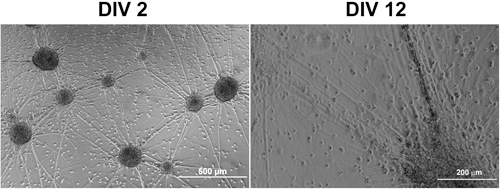
 **Figure 2.**  Phase contrast images of an aggregate culture 2 and 12 days after seeded on Matrigel-coated coverslips.

## Discussion

Early studies reported formation of synapses and mature myelin in rotation-mediated free floating aggregate cultures^1^. The CNS aggregate culture system described here combines serum-free growth of multipotent progenitor cells in three dimensional aggregates with the convenience of traditional 2D cultures to facilitate analyses of cell development, migration and differentiation *in vitro.* The system can be modified and used for neural precursor cell research and for investigation into neuron-glia interactions. For example, genetically modified cells such as oligodendrocyte progenitors^2^ may be added to the aggregate cultures. Effect of immune cells such as microglia and macrophages or various reagents on the development and differentiation of neurons and glia can be also studied. Reaggregates of purified retinal ganglion cells have recently been used to study CNS myelination in cocultures with oligodendrocytes in the absence of other cells^3^. In agreement with previous report^4^, Matrigel appears to be superior to PDL in that it promotes cell adhesion and accelerates neurite outgrowth and glia differentiation. Matrigel is therefore used as the substratum for cell aggregates in this protocol. 

## References

[B0] Matthieu JM (1978). Myelination in rat brain aggregating cell cultures. Neuroscience.

[B1] Chen Ying (2007). Isolation and culture of rat and mouse oligodendrocyte precursor cells. Nat. Protocols.

[B2] Watkins TA, Emery B, Mulinyawe S, Barres BA (2008). Distinct stages of myelination regulated by gamma-secretase and astrocytes in a rapidly myelinating CNS coculture system. Neuron.

[B3] Svenningsen AF, Shan Wei-Song, Colman DavidR, Pedraza Liliana (2003). Rapid method for culturing embryonic neuron-glial cell cocultures. Journal of Neuroscience Research.

